# Breast cancer chemotherapy in transition: predictive markers, resistance mechanisms, and new treatment approaches

**DOI:** 10.3389/fonc.2026.1856398

**Published:** 2026-05-08

**Authors:** Rakesh Sharma, Leelabati Toppo, Prakasini Satapathy, Rekha Arcot, Chandana Maji, Sorabh Lakhanpal, Sanjay Kumar, Do-Youn Lee

**Affiliations:** 1 Department of Biochemistry, Graphic Era Deemed University, Dehradun, India; 2 Centre for Promotion of Research, Graphic Era Hill University, Dehradun, India; 3 Department of General Medicine, Malla Reddy Medical College for Women, Telangana, India; 4 School of Medical Sciences and Research, Sharda University, Greater Noida, India; 5 Department of surgery, Dr. D. Y. Patil Medical College, Hospital and Research Centre, Dr. D. Y. Patil Vidyapeeth (Deemed-to-be-University), Maharashtra, India; 6 Noida Institute of Engineering & Technology (Pharmacy Institute), Greater Noida, India; 7 School of Pharmaceutical Sciences, Lovely Professional University, Phagwara, India; 8 Centre for Research Impact & Outcome, Chitkara College of Pharmacy, Chitkara University, Punjab, India; 9 College of General Education, Kookmin University, Seoul, Republic of Korea

**Keywords:** breast cancer, chemotherapy, drug resistance, neoadjuvant therapy, predictive biomarkers, therapeutic strategies

## Abstract

Breast cancer remains the most frequently diagnosed malignancy among women worldwide and continues to be a major cause of cancer-related mortality despite substantial therapeutic progress. Chemotherapy remains a central component of treatment, especially in high-risk early breast cancer, triple-negative breast cancer (TNBC), HER2-positive disease, and selected hormone receptor-positive tumors. Over the last two decades, the role of chemotherapy in breast cancer has evolved from a largely uniform cytotoxic approach to a more nuanced strategy guided by tumor biology, response dynamics, and residual disease burden. Neoadjuvant chemotherapy has become particularly important, not only for tumor downstaging but also for providing an *in vivo* test of chemosensitivity, with pathologic complete response (pCR) serving as a useful marker of long-term outcome in aggressive biological subtypes. At the same time, increasing knowledge of predictive biomarkers such as BRCA1/2 status, homologous recombination deficiency (HRD), tumor-infiltrating lymphocytes (TILs), Ki-67, intrinsic molecular subtypes, and circulating tumor DNA (ctDNA) has improved patient stratification and opened opportunities for more personalized treatment. However, chemotherapy resistance remains a major obstacle. Resistance arises through multiple mechanisms, including drug efflux, enhanced DNA repair, apoptotic dysregulation, epithelial-mesenchymal transition, cancer stem cell plasticity, metabolic adaptation, and tumor microenvironment-mediated immune suppression. These mechanisms limit durable response and contribute to relapse, particularly in TNBC and residual disease after neoadjuvant therapy. Recent therapeutic advances have focused on overcoming resistance through platinum agents, PARP inhibitors, immune checkpoint blockade, post-neoadjuvant escalation with capecitabine or trastuzumab emtansine, and emerging antibody-drug conjugates. This review summarizes the advancement of chemotherapy in breast cancer.

## 1 Introduction

Breast cancer is a biologically heterogeneous disease encompassing multiple histologic and molecular subtypes with distinct clinical behaviors and therapeutic vulnerabilities. Although endocrine therapy, HER2-directed therapy, and other targeted approaches have transformed management, chemotherapy remains indispensable in many patients, especially those with TNBC, HER2-positive tumors receiving combination treatment, and high-risk luminal disease (1). The historical development of chemotherapy in breast cancer was driven by anthracyclines and taxanes, which improved response rates and survival in both adjuvant and neoadjuvant settings. Over time, the neoadjuvant platform has become particularly valuable because it allows direct assessment of tumor response and supports adaptive treatment strategies based on residual disease (2, 3).

The concept of pCR has significantly shaped modern breast cancer chemotherapy research. Analyses from large pooled datasets showed that pCR is associated with favorable long-term outcomes, especially in TNBC and HER2-positive disease, though its predictive strength varies across subtypes (4, 5). This has encouraged the incorporation of biomarker-informed neoadjuvant regimens and has promoted pCR as an endpoint in clinical trials evaluating treatment intensification or de-escalation (6, 7).

Despite these advances, the benefits of chemotherapy are not uniform. Many tumors demonstrate primary refractoriness, and others develop acquired resistance during treatment. This challenge has intensified interest in predictive biomarkers and in understanding resistance mechanisms at genomic, transcriptomic, epigenetic, and microenvironmental levels (8). A more precise understanding of response determinants is essential to reduce overtreatment, avoid unnecessary toxicity, and improve survival outcomes.

This mini review discusses the advancement of chemotherapy in breast cancer through three interrelated lenses: predictive markers of treatment response, mechanisms of chemoresistance, and therapeutic strategies designed to overcome resistance and optimize care.

## 2 Methods

This mini-review was designed as a focused narrative synthesis rather than a systematic review. Literature was identified primarily through PubMed/Medline and Google Scholar, with emphasis on English-language studies published from approximately 2000 to 2025 that addressed neoadjuvant and adjuvant chemotherapy, predictive biomarkers of response, mechanisms of resistance, and therapeutic strategies to overcome resistance in breast cancer. Priority was given to landmark randomized trials, pooled analyses, meta-analyses, practice-informing biomarker studies, and selected translational research with particular relevance to early-stage TNBC, HER2-positive disease, and high-risk HR-positive/HER2-negative breast cancer. In areas where evidence was rapidly evolving or overlapping, including antibody-drug conjugates, ctDNA, and response-adapted post-neoadjuvant strategies, we preferentially emphasized studies with the greatest clinical interpretability, practice relevance, or conceptual importance rather than attempting exhaustive coverage of all emerging reports. The aim was not comprehensive enumeration of the literature, but a clinically oriented synthesis of evidence most relevant to the evolving role of chemotherapy in modern breast oncology.

## 3 Evolution of chemotherapy in breast cancer

Chemotherapy in breast cancer has evolved from empiric use of cytotoxic agents to increasingly biology-guided treatment. Early neoadjuvant studies established the feasibility and clinical benefit of preoperative chemotherapy. The NSABP B-18 trial demonstrated that preoperative chemotherapy could achieve tumor downstaging and increase rates of breast-conserving surgery without compromising survival compared with postoperative delivery (3). The NSABP B-27 trial further showed that adding docetaxel after doxorubicin and cyclophosphamide increased clinical and pathological response rates, strengthening the role of sequential anthracycline-taxane regimens (2).

Subsequent pooled analyses refined the clinical meaning of response. The CTNeoBC collaboration confirmed that pCR correlates with improved event-free and overall survival, especially in biologically aggressive subtypes such as TNBC and HER2-positive/hormone receptor-negative disease (4). Similar findings from other analyses reinforced the prognostic relevance of pCR but also highlighted that it is not an equally reliable surrogate across all breast cancer subtypes (5, 6). More recent meta-analytic evidence continues to support pCR as a useful response endpoint while emphasizing the need to interpret it in the context of intrinsic subtype and treatment platform (7).

This evolving framework shifted chemotherapy from a one-size-fits-all intervention toward a platform for precision escalation, response-guided adaptation, and biomarker discovery. In current practice, chemotherapy is no longer viewed solely as a systemic cytotoxic backbone but as part of a larger therapeutic architecture that includes predictive biomarkers, targeted agents, and post-neoadjuvant decision-making.

## 4 Predictive markers of chemotherapy response

### 4.1 Pathologic complete response as a response marker

Among predictive and prognostic tools, pCR remains one of the most widely studied endpoints in breast cancer treated with neoadjuvant chemotherapy. Achievement of pCR is associated with improved long-term outcomes, particularly in TNBC and HER2-positive disease, where chemosensitivity is generally higher and residual invasive disease carries substantial recurrence risk. However, the meaning of pCR requires careful interpretation. At the patient level, pCR is a strong favorable prognostic marker in aggressive subtypes. At the trial level, however, increases in pCR do not always translate proportionally into improved event-free or overall survival, meaning that pCR is an imperfect surrogate endpoint for treatment benefit across all settings. This distinction is especially important in luminal tumors, where pCR rates are lower and prognosis is influenced not only by chemotherapy response but also by endocrine sensitivity and underlying tumor biology. Accordingly, pCR is best viewed not as a universal surrogate for benefit, but as a subtype-dependent response marker that is most clinically informative in TNBC and HER2-positive disease and less definitive in HR-positive/HER2-negative tumors (4–7).

### 4.2 BRCA1/2 mutations and homologous recombination deficiency

DNA repair deficiency has emerged as one of the most clinically actionable predictive domains in breast cancer. Tumors harboring germline BRCA1/2 mutations often exhibit heightened sensitivity to DNA-damaging agents, particularly platinum salts and PARP inhibition, due to defective homologous recombination repair. Studies evaluating HRD scores have shown that genomic scarring and homologous recombination dysfunction are associated with better response to platinum-containing neoadjuvant chemotherapy in TNBC and BRCA-associated disease (9, 10). These findings have helped position HRD as a promising biomarker for therapy selection, although standardization of testing remains a challenge.

The biological rationale is strong: tumors unable to accurately repair double-strand DNA breaks are more vulnerable to agents such as carboplatin that induce DNA crosslinking and genomic instability. This principle has also supported the clinical use of adjuvant olaparib in high-risk germline BRCA-mutated early breast cancer after local treatment and chemotherapy (11).

### 4.3 Tumor-infiltrating lymphocytes

The immune microenvironment is increasingly recognized as a determinant of chemotherapy response. TILs are particularly relevant in TNBC and HER2-positive disease, where higher baseline stromal lymphocytic infiltration has been associated with increased pCR rates and improved survival (12). Meta-analytic data also suggest that TILs possess both prognostic and predictive significance in patients receiving neoadjuvant chemotherapy (13). This may reflect greater immune activation, higher neoantigen burden, or immunogenic cell death induced by cytotoxic therapy. The integration of TIL assessment into routine pathology remains variable, but it has become an important translational biomarker.

### 4.4 Intrinsic molecular subtype and gene-expression signatures

Gene-expression profiling has refined breast cancer classification beyond routine immunohistochemistry. The work of Sotiriou and Pusztai (2009) highlighted the importance of genomic signatures in predicting prognosis and therapeutic sensitivity (1). Intrinsic subtyping systems, including PAM50, distinguish luminal A, luminal B, HER2-enriched, and basal-like tumors, each with different expected chemotherapy responsiveness. For example, HER2-enriched tumors often show robust response to anti-HER2 plus chemotherapy regimens, whereas luminal A tumors tend to derive less benefit from cytotoxic intensification (14). Molecular profiling thus supports better selection of patients likely to benefit from chemotherapy. Although chemotherapy is most strongly emphasized in TNBC and HER2-positive disease, decision-making has also evolved substantially in HR-positive/HER2-negative breast cancer. In this setting, chemotherapy benefit is heterogeneous and depends on tumor burden, nodal status, grade, menopausal context, and biological subtype. Luminal A tumors generally have lower chemosensitivity and derive less benefit from cytotoxic intensification, whereas luminal B tumors, which are more proliferative and biologically aggressive, are more likely to warrant chemotherapy in addition to endocrine therapy. This distinction is important because a broad discussion of chemotherapy in breast cancer should not imply that all HR-positive disease behaves similarly; rather, the role of chemotherapy in luminal tumors is increasingly selective and biology-informed.

### 4.5 Ki-67 and proliferation-associated markers

Ki-67 remains a practical but imperfect marker of tumor proliferation. High Ki-67 expression is often associated with greater chemotherapy responsiveness because rapidly proliferating tumors are generally more chemosensitive, but it also reflects more aggressive disease biology. Its clinical value is therefore context dependent. In routine practice, Ki-67 may contribute to risk stratification, particularly in HR-positive disease, but its role as a stand-alone biomarker for chemotherapy selection remains limited by assay variability, differences in cutoffs, and interobserver inconsistency. Thus, Ki-67 is better regarded as a supportive marker within broader clinicopathologic and molecular assessment rather than a definitive chemotherapy-directing biomarker.

### 4.6 Circulating tumor DNA

ctDNA is emerging as a dynamic biomarker for treatment response monitoring and minimal residual disease detection. Persistent or re-emergent ctDNA after neoadjuvant therapy may indicate residual resistant disease and increased relapse risk, whereas ctDNA clearance may reflect effective treatment response. Nevertheless, ctDNA remains primarily an investigational tool in routine chemotherapy decision-making for early breast cancer. Key challenges include assay standardization, sensitivity in low-volume disease, optimal sampling time points, and uncertainty about how ctDNA-guided treatment changes should be implemented prospectively. At present, ctDNA is best viewed as a highly promising translational biomarker with potential future utility in response-adapted escalation or de-escalation, rather than an established standard-of-care determinant of chemotherapy selection. A summary of the major predictive biomarkers of chemotherapy response is provided in **Table 1**.

**Table 1:** Predictive markers of chemotherapy response in breast cancer.

Predictive marker	Biological relevance	Clinical significance in chemotherapy	Clinical readiness/current practice relevance	Key supporting references
Pathologic complete response (pCR)	Reflects eradication of invasive tumor after neoadjuvant therapy	Associated with improved event-free and overall survival, especially in TNBC and HER2-positive disease	Established, but interpretation is subtype-dependent; more clinically informative in TNBC and HER2-positive disease than in HR-positive/HER2-negative tumors	(Cortazar et al., 2014; Spring et al., 2020; von Minckwitz et al., 2012) (4, 5, 7)
BRCA1/2 mutation	Defective homologous recombination DNA repair	Predicts sensitivity to platinum agents and PARP inhibitors	Clinically actionable in relevant treatment settings	(Telli et al., 2017; Telli et al., 2016; Tutt et al., 2021) (9–11)
Homologous recombination deficiency (HRD)	Genomic instability and impaired double-strand DNA repair	May identify tumors more likely to respond to platinum-based chemotherapy	Promising, but not uniformly standardized for routine chemotherapy decision-making	(Telli et al., 2017; Telli et al., 2016) (9, 10)
Tumor-infiltrating lymphocytes (TILs)	Reflect host antitumor immune response	Higher TIL levels correlate with better pCR and prognosis, especially in TNBC	Translationally valuable; limited routine stand-alone treatment-directing role	(Dieci et al., 2014; Li et al., 2022) (12, 13)
Intrinsic molecular subtype (e.g., PAM50)	Defines tumor biology beyond routine histopathology	Helps predict chemosensitivity and outcome across breast cancer subtypes	Clinically informative for treatment context and expected chemotherapy sensitivity	(Prat et al., 2015; Sotiriou & Pusztai, 2009) (1, 14)
Ki-67	Marker of tumor proliferation	High expression often linked with greater chemotherapy responsiveness but also aggressive behavior	Supportive and context-dependent; not a definitive stand-alone chemotherapy-directing biomarker	(Spring et al., 2020; von Minckwitz et al., 2012) (5, 7)
Circulating tumor DNA (ctDNA)	Dynamic marker of residual disease and treatment response	May help identify nonresponders and patients at high risk of relapse	Investigational for routine chemotherapy decision-making in early breast cancer	(Spring et al., 2020) (7)

## 5 Mechanisms of chemotherapy resistance

Resistance to chemotherapy is multifactorial and often involves overlapping tumor-intrinsic and microenvironmental processes. In breast cancer, the clinical relevance of these mechanisms lies not only in explaining treatment failure but also in shaping biomarker development, residual disease interpretation, and rational combination strategies. Accordingly, resistance biology is most useful when linked to specific therapeutic implications rather than viewed only as a broad catalogue of cellular adaptations.

### 5.1 Drug efflux and reduced intracellular drug accumulation

One of the classic mechanisms of resistance is increased expression of ATP-binding cassette transporters such as P-glycoprotein, which actively export chemotherapeutic agents from cancer cells. This reduces intracellular drug concentrations and diminishes efficacy, particularly for taxanes and anthracyclines. Drug transporter-mediated resistance remains an important component of multidrug resistance phenotypes (15).

### 5.2 Enhanced DNA repair

DNA repair proficiency is a major determinant of chemotherapy sensitivity. Tumors that restore homologous recombination capacity or upregulate compensatory DNA damage response pathways may become less responsive to platinum agents and less vulnerable to PARP-directed strategies (8, 11). This is particularly relevant in BRCA-associated and HRD-high tumors, where initial vulnerability may be followed by adaptive resistance (8, 9, 11). Clinically, this has implications for patient selection, interpretation of residual disease, and the design of combination strategies that target DNA damage response signaling (9, 11).

### 5.3 Apoptotic dysregulation

Even when chemotherapy induces DNA damage or mitotic stress, tumor cells may survive if apoptotic pathways are impaired. Alterations in TP53, overexpression of anti-apoptotic proteins, and disruption of mitochondrial death signaling all contribute to chemoresistance. In aggressive subtypes such as TNBC, apoptotic dysregulation frequently coexists with genomic instability, creating a paradox of both therapeutic vulnerability and resistance potential.

### 5.4 Cancer stem cells and phenotypic plasticity

Cancer stem-like cells represent a small but clinically important therapy-tolerant population capable of self-renewal, tumor repopulation, and relapse. In breast cancer, these cells may persist despite reduction of the bulk tumor during neoadjuvant treatment and may contribute to residual disease after apparently effective chemotherapy. Their relevance is therefore not only biological but also translational: stemness-related persistence helps explain why some patients fail to convert pCR gains into durable disease control and supports interest in post-neoadjuvant escalation and stemness-targeted investigational strategies (8).

### 5.5 Epithelial-mesenchymal transition

Epithelial-mesenchymal transition (EMT) promotes invasiveness, motility, and resistance by reprogramming tumor cells toward a mesenchymal phenotype. EMT is associated with stemness features, immune evasion, and decreased susceptibility to chemotherapy-induced cell death. It is strongly influenced by the tumor microenvironment, including hypoxia, inflammatory cytokines, and stromal interactions. EMT-mediated resistance is especially relevant in TNBC, where mesenchymal signaling programs are often enriched (8).

### 5.6 Metabolic reprogramming

Resistant tumor cells can adapt their metabolism to survive therapeutic stress. Altered glycolysis, mitochondrial rewiring, lipid metabolism, and redox homeostasis have all been implicated in resistance to anthracycline-taxane combinations. Metabolic plasticity supports survival under oxidative stress and may allow tumor cells to endure cytotoxic exposure until selective pressure is relieved. Studies have linked resistant breast tumors to inflammatory and metabolic signatures, reinforcing the concept that resistance is not exclusively genomic (16).

### 5.7 Tumor microenvironment and immune-mediated resistance

The tumor microenvironment, including fibroblasts, immune cells, extracellular matrix components, and soluble mediators, can protect tumor cells from chemotherapy. Immunosuppressive macrophages, regulatory T cells, stromal remodeling, and cytokine networks may blunt effective antitumor immunity or create physical barriers to drug penetration. Since part of chemotherapy efficacy may depend on immunogenic cell death, a suppressive microenvironment can meaningfully reduce therapeutic benefit.

## 6 Therapeutic strategies to overcome resistance

### 6.1 Platinum incorporation in triple-negative breast cancer

The addition of platinum agents to neoadjuvant chemotherapy has been one of the most important recent developments in TNBC (17–19). Trials such as CALGB 40603 and GeparSixto showed that carboplatin can improve pCR rates when added to anthracycline-taxane-based regimens (18, 19). However, the clinical interpretation is more nuanced than pCR improvement alone. Long-term survival benefit has been less consistent across studies and settings, and treatment intensification comes with meaningful hematologic toxicity, greater treatment burden, and practical patient-selection challenges (17–19). These considerations are especially relevant because not all TNBCs derive the same magnitude of benefit. Platinum appears most compelling in tumors with BRCA mutations or broader homologous recombination deficiency features, supporting a biomarker-enriched rather than uniformly intensified approach (9, 10, 17).

### 6.2 PARP inhibition in DNA repair-deficient disease

PARP inhibitors exploit synthetic lethality in tumors with defective homologous recombination. The OlympiA trial established that adjuvant olaparib significantly improves invasive disease-free survival and overall survival in patients with high-risk HER2-negative early breast cancer harboring germline BRCA1/2 mutations after local therapy and chemotherapy (11). This represents a major shift in post-chemotherapy management and highlights the importance of germline testing in appropriate patients.

### 6.3 Immunochemotherapy

Immune checkpoint inhibitors have expanded the role of chemotherapy by enhancing antitumor immune responses. In KEYNOTE-522, the addition of pembrolizumab to neoadjuvant chemotherapy improved pCR and event-free survival in early TNBC, establishing immunochemotherapy as a new standard in many high-risk cases (20). Chemotherapy may promote antigen release and immune activation, thereby sensitizing tumors to checkpoint blockade. This strategy is particularly compelling in tumors with higher TIL levels and more inflamed microenvironments.

### 6.4 Post-neoadjuvant escalation for residual disease

Residual disease after neoadjuvant chemotherapy is a strong marker of poor prognosis and treatment resistance. This has led to effective post-neoadjuvant escalation strategies. The CREATE-X trial showed that adjuvant capecitabine improved outcomes in patients with residual invasive HER2-negative breast cancer after standard neoadjuvant chemotherapy, with notable benefit in TNBC (21). Similarly, the KATHERINE trial demonstrated that switching to trastuzumab emtansine significantly reduced invasive disease recurrence in patients with residual HER2-positive disease after neoadjuvant therapy (22). These studies illustrate how response-adapted therapy can convert residual disease information into actionable treatment decisions.

### 6.5 Antibody-drug conjugates and emerging approaches

Antibody-drug conjugates (ADCs) represent one of the clearest examples of how chemotherapy is being re-engineered rather than simply intensified (23). Conceptually, ADCs occupy an important middle ground between conventional cytotoxic therapy and precision oncology: they retain the antitumor potency of chemotherapy but deliver the cytotoxic payload through antigen-directed targeting, thereby potentially improving therapeutic selectivity (23). In this sense, ADCs are highly relevant to the future of breast cancer chemotherapy even when their current established roles vary by subtype and disease setting (23–26).

Their clinical importance is already well recognized in breast cancer, particularly in HER2-expressing disease and, increasingly, in other biologically defined subsets (22, 24–26). Although much of the mature evidence for ADCs has emerged in advanced or metastatic settings, their broader significance for this review lies in the way they redefine what “chemotherapy” may mean in contemporary breast oncology (23–26). Rather than relying solely on nonselective systemic exposure, ADCs illustrate a shift toward targeted cytotoxic delivery linked to tumor biology (23). This is especially relevant in a review focused on predictive markers and resistance, because ADC development depends on biomarker-defined targeting, may partially circumvent some forms of conventional drug resistance, and reflects the convergence of chemotherapy with molecular stratification (23).

At the same time, ADCs should still be discussed with appropriate caution. Their activity does not eliminate the challenges of resistance, toxicity, or patient selection, and their precise role in early breast cancer continues to evolve (22, 23). For that reason, ADCs are best viewed not simply as another drug class, but as a therapeutic model that may increasingly reshape how cytotoxic treatment is integrated into subtype-specific breast cancer care (23). A schematic overview of resistance mechanisms and corresponding treatment strategies is presented in **Table 2**.

**Table 2:** Major mechanisms of chemotherapy resistance and therapeutic strategies in breast cancer.

Resistance mechanism	Underlying process	Clinical consequence	Therapeutic strategy to overcome resistance	Clinical readiness/current therapeutic relevance	Key supporting references
Drug efflux transporter overexpression	Increased export of cytotoxic drugs from tumor cells	Reduced intracellular drug concentration and multidrug resistance	Development of transporter-targeted approaches and alternative drug platforms	Mechanistically established, but with limited direct routine targeting in current practice	(Tufail et al., 2022) (8)
Enhanced DNA repair	Efficient repair of chemotherapy-induced DNA damage	Reduced sensitivity to anthracyclines and platinum agents	Platinum selection in HRD tumors; PARP inhibitors in BRCA-mutated disease	Clinically relevant, especially through platinum/PARP-linked treatment strategies	(Telli et al., 2016; Tutt et al., 2021) (9, 11)
Apoptotic dysregulation	Impaired cell death signaling after chemotherapy injury	Tumor survival despite cytotoxic damage	Combination regimens and targeted therapies aimed at vulnerable signaling pathways	Translationally important, but not routinely targetable in standard care	(Tufail et al., 2022) (8)
Cancer stem cell persistence	Survival of self-renewing, therapy-tolerant cell populations	Relapse and disease recurrence	Investigational stemness-targeted and post-neoadjuvant escalation strategies	Investigational, but relevant to residual disease biology and relapse risk	(Tufail et al., 2022) (8)
Epithelial-mesenchymal transition (EMT)	Increased plasticity, invasion, and resistance phenotype	Poor response and metastatic progression	Combination strategies targeting microenvironment and resistant residual disease	Translationally important; indirect therapeutic relevance at present	(Menyhárt et al., 2024; Tufail et al., 2022) (8, 16)
Metabolic rewiring	Adaptive changes in energy production and oxidative stress handling	Survival under chemotherapy pressure	Emerging metabolism-targeted strategies	Emerging investigational relevance	(Menyhárt et al., 2024) (16)
Immune-suppressive tumor microenvironment	Reduced antitumor immune activity and impaired immunogenic cell death	Lower treatment response and persistent residual disease	Chemo-immunotherapy with pembrolizumab in TNBC	Clinically relevant in immunochemotherapy settings	(Schmid et al., 2022) (20)
Residual disease after neoadjuvant chemotherapy	Indicates persistence of resistant tumor clones	Higher risk of recurrence	Capecitabine in HER2-negative residual disease; T-DM1 in HER2-positive residual disease	Established basis for post-neoadjuvant treatment escalation	(Masuda et al., 2017; von Minckwitz et al., 2019) (21, 22)
HRD/BRCA-associated vulnerability	Defect in homologous recombination repair	Selective treatment sensitivity rather than pure resistance	Platinum-based regimens; adjuvant olaparib	Clinically relevant, especially in biomarker-selected populations	(Loibl et al., 2018; Tutt et al., 2021) (11, 17)

## 7 Future directions

The advancement of chemotherapy in breast cancer is moving toward personalization rather than abandonment. Chemotherapy will likely remain essential for many patients, but its delivery is becoming more selective, adaptive, and biologically integrated. Future progress will depend on refining predictive markers, especially through multimodal approaches that combine genomic alterations, immune contexture, molecular subtyping, and dynamic biomarkers such as ctDNA. Better identification of true chemosensitive disease could minimize toxicity in low-benefit populations while enabling rational escalation in high-risk groups.

At the same time, overcoming resistance will require moving beyond single-marker thinking. Resistance is rarely explained by one pathway alone; instead, it reflects evolving tumor ecosystems. Integrative profiling before, during, and after treatment may allow clinicians to identify emerging resistant clones and modify therapy accordingly. The neoadjuvant setting remains ideal for this research because it offers serial tissue access and direct assessment of response.

Finally, future therapeutic strategies will likely blend chemotherapy with immunotherapy, targeted agents, and post-neoadjuvant residual disease–directed interventions. Emerging platforms such as antibody-drug conjugates further suggest that the future of chemotherapy may depend less on uniform intensification and more on biologically targeted cytotoxic delivery, particularly as subtype-specific treatment architectures become more refined. In this sense, the modern question is no longer whether chemotherapy still matters in breast cancer, but how it can be deployed more intelligently within precision oncology.

## 8 Conclusion

Chemotherapy remains a cornerstone of breast cancer treatment, but its role is now increasingly defined by who benefits, in which subtype, and at what treatment stage, rather than by uniform use across all patients. Its modern value is greatest in biologically aggressive and high-risk settings, especially TNBC, HER2-positive disease treated in combination with HER2-directed therapy, and selected HR-positive/HER2-negative tumors with higher-risk biological features. Advances in neoadjuvant therapy, residual disease assessment, and biomarker-guided escalation have made chemotherapy more informative as well as more selective.

At the same time, substantial challenges persist due to multidimensional mechanisms of resistance involving drug efflux, enhanced DNA repair, apoptotic escape, EMT, cancer stemness, metabolic adaptation, and tumor microenvironmental protection. Importantly, these insights have already produced meaningful therapeutic gains, including platinum-based intensification, PARP inhibition, immunochemotherapy, and post-neoadjuvant escalation with capecitabine or trastuzumab emtansine.

For clinicians, the key implication is that chemotherapy should increasingly be integrated with subtype, biomarker context, and response dynamics rather than prescribed as a one-size-fits-all backbone; for researchers, the priority is to refine which markers are truly actionable, how resistance can be intercepted earlier, and how post-neoadjuvant strategies can be matched more precisely to residual risk.
